# Dynamic Quality of Service Model for Improving Performance of Multimedia Real-Time Transmission in Industrial Networks

**DOI:** 10.1371/journal.pone.0105885

**Published:** 2014-08-29

**Authors:** Ravichandran C. Gopalakrishnan, Manivannan Karunakaran

**Affiliations:** 1 SCAD Institute of Technology, Palladam, Tiruppur, Tamilnadu, India; 2 Department of Computer Science and Engineering, PSNA College of Engineering and Technology, Dindigul, Tamilnadu, India; Tianjin University of Technology, China

## Abstract

Nowadays, quality of service (QoS) is very popular in various research areas like distributed systems, multimedia real-time applications and networking. The requirements of these systems are to satisfy reliability, uptime, security constraints and throughput as well as application specific requirements. The real-time multimedia applications are commonly distributed over the network and meet various time constraints across networks without creating any intervention over control flows. In particular, video compressors make variable bit-rate streams that mismatch the constant-bit-rate channels typically provided by classical real-time protocols, severely reducing the efficiency of network utilization. Thus, it is necessary to enlarge the communication bandwidth to transfer the compressed multimedia streams using Flexible Time Triggered- Enhanced Switched Ethernet (FTT-ESE) protocol. FTT-ESE provides automation to calculate the compression level and change the bandwidth of the stream. This paper focuses on low-latency multimedia transmission over Ethernet with dynamic quality-of-service (QoS) management. This proposed framework deals with a dynamic QoS for multimedia transmission over Ethernet with FTT-ESE protocol. This paper also presents distinct QoS metrics based both on the image quality and network features. Some experiments with recorded and live video streams show the advantages of the proposed framework. To validate the solution we have designed and implemented a simulator based on the Matlab/Simulink, which is a tool to evaluate different network architecture using Simulink blocks.

## Introduction

Distributed enterprise networks have the capacity to transmit any number of multimedia files to any number of channel servers without any special setup. This means the channels added in the network will be received as video.

Video compression is performed to change the bit rate of the videos from constant to variable. Classical network communication protocols allow only videos with constant bit rate and none with variable bit rates. Some automation is needed to change the bandwidth and adapt to a variable bit rates. Aspects of network performance that are often captured in QoS measures include availability (uptime), bandwidth (throughput), latency (delay), and error rate [1], [12], [22]. Depending upon a server or router's performance, QoS parameters are used to identify network interfaces for particular applications. QoS is especially important for the new generation of Internet applications. However, ethernet was not designed to support accurate performance while implementing QoS solutions across the Internet. Typically, new generation applications are complex and heterogeneous, encompassing several real-time activities in addition to media processing. Thus, any interference caused by or to multimedia-handling components must be limited and predictable.

The distribution of media control applications (MCAs) such as object tracking, automated inspection, machine vision [2] and vehicle guidance in industry continues to increase. Industrial MCAs fall into two broad classes [1]: supervised multimedia control subsystems [8] and multimedia embedded Systems (MESs). In the first class, emphasis is given to media processing quality, with little real-time constraints. Whereas MESs are more demanding, requiring both processing quality and stringent real-time requirements. Typically, these applications are complex and heterogeneous, encompassing several real-time activities in addition to media processing. As a result it is necessary to limited and predict any interference caused to (and suffered by) the multimedia handling components. Many MES applications are distributed, relying on real-time network protocols to provide the necessary real-time communication services. However, multimedia traffic (especially video streaming) has specific characteristics that conflict with the operational framework of conventional real-time protocols. Specifically, due to the types of compressor used, multimedia information exists as a variable bit-rate(VBR) traffic source, whereas the real-time networks typically offer applications constant-bit-rate (CBR) channels (e.g., PROFINET-IRT, ATM, ControlNet, Interbus, or flexible time triggered FTT networks)[9]–[12]. Matching a VBR source to a CBR channel is non-trivial and can lead to waste of bandwidth or rejection of frames. This difficulty became particularly challenging with the emergence of MES applications that impose reliability and timeliness requirements that cannot be fulfilled by standard network protocols [13], usually due to a lack of temporal isolation and consequent unbounded mutual interference between streams.

We proposed dynamic QoS management features of the FTT over Switched Ethernet protocol (FTT-SE) to carry out the referred adaptation with MJPEG video streams. In particular, we proposed managing in an integrated way both the compression parameters and the frame acquisition period, which drive the encoding of the bit rate that must fit strictly inside the network bandwidth allocated to each stream. On the other hand, the channel bandwidth, by means of the frame transmission period, is adapted on-line according to the current needs of the whole set of channels currently supported by the system. These needs take into consideration the set up and tear down of channels as well as structural changes within each stream. This approach facilitates the compression procedure, and attempts to maximize the QoS level provided by each channel considering the current total bandwidth requirements, thus providing an efficient way to share the network bandwidth among different streams. We present four major methodologies in this paper for improving the average performance of real time communication over switched ethernet supporting QoS management by using FTT-ESE.

First, in addition to the channel period, the QoS layer can now also adapt the channel width (*C*), thus enlarging the configuration space. This approach brings higher flexibility and increased granularity to the channel bandwidth allocation mechanism. This feature is of high practical relevance, since most of the imaging devices restrict the frame acquisition periods to discrete predefined sets, resulting in a correspondingly discrete stepwise bandwidth allocation function that limits the configuration space options. Therefore, adjusting the channel width and period together allows gaining smooth mode changes and provides a much richer configuration space. In addition, for some systems, changing the channel period is undesirable due to rate, coupling among different channels or to constraints imposed by application-level controllers (e.g., sample period). In this case, the only possibility is to adapt the channel width while keeping the respective period constant. Second, the work presented in this paper is using a richer set of inputs to the QoS master. In particular, the computation of the system benefit takes into account the image quality, as well as the bandwidth usage, allowing different benefit/cost tradeoffs. This paper is also presents significant experimental results by including tests using more streams, with more dynamic requirements, in more scenarios and for longer duration. Finally, the description of the state of the art has also been significantly enhanced.

The contribution of the proposed work is as follows:

- We extend a FTT-ESE protocol for providing an automatic process to calculate the compression level and change the bandwidth of the stream.
- We propose a dynamic QOS management to increase the automation process of recomposing the compressed rates and bandwidth allocation is established.
- We enhance the QoS negotiation algorithm and Bandwidth distribution algorithms provide the significant results in the multimedia real-time transmission.
- We present four static and dynamic streams in the proposed framework and achieved 90% of the efficient QoS levels in different QoS features.
- We have designed and implemented a simulator based on the Matlab/Simulink which is a tool to evaluate different network architecture using Simulink blocks.

This paper is organized in the following way. The next section presents a review of the related works. Section 3 describes the system model while section 4 discusses an experimental environment, presenting a set of experiments carried out to assess to perform the method proposed. Section 5 concludes the paper and presents the future work.

## Related Works

Multimedia compression standards [10] and multimedia transmission [1] are used to identify redundant data, e.g., groups of pixels of similar color, to reduce the data size. Different compression standards (**Table 1**), such as JPEG (with baseline, progressive, hierarchical and lossless profiles) [2], JPEG2000 [21], MPEG-2 [1], H.263 [3] and MPEG-4 (part 10 [5], also known as H.264 or MPEG-4 AVC) allow the system to cope with the requirements posed by the different classes of applications.Two main types of compression are applied during transmission: image compressors and video compressors. An image compressor compresses between frames, while a video compressor uses the redundancy of images in a sequential manner. Depending on the application being used, one must choose the compression technique. In recent years, many image compressor standards like JPEG and video compressor standards like MPEG [3], [17] are applied during multimedia transmission. In contrast, video coding is a compression technique that is performed by the application. The result is higher compression rates, which use less bandwidth during transmission when the load of the network can be affected by various parameters. The video coding technique maintains image quality at a constant rate. This is not suitable for industrial applications where the image quality changes often. In image compression, all images are independent, so if any, loss occurs in one image during transmission, nothing affects the subsequent images being transmitted. In turn, video transmission uses different frame types, namely, I-frames, which are independent, but also P-frames, i.e., inter-frames coded depending on previous frames, and sometimes B-frames that depend on following frames. Only I-frames are self-contained; thus, the loss of a frame or part of it may have an impact on several of the following frames, until the arrival of another I-frame.But in another important characteristic of video transmission in industrial applications is that the images of different streams are sometimes captured at low rates and multiplexed together in the same channel.In this situation the compression level is redundant.

**Table 1:** Properties of encoders.

Property	MJPEG	MJPEG2000	MJPEG4-Part 10
VBR Support	Yes	Yes	Yes
CBR Support	No	Yes	Yes
Latency	Low	Low to Medium	High
Motion Compensation	No	No	Yes
Relative Cost	1x	3x	2x

In recent years, multimedia real-time transmission over the Internet [16], [18] has generated a significant amount of research interest. Typical solutions have been based on the transmission control protocol (TCP), the user datagram protocol (UDP), or the Internet protocol (IP) stack, complemented by other protocols, such as real-time protocol (RTP)/real-time control protocol (RTCP), real-time streaming protocol (RTSP), or session initiation protocol (SIP). These protocols measure key network parameters such as bandwidth usage, packet loss rate, and round-trip delays to control the load put on the network. The calculation of the required bandwidth for storing video as well as live video (video streams).The efficiency of the quality results is highly dependent on the delay allowed by the particular application. This is one of the main limitations of these technologies. Some of the algorithms presented in Pedreiras et al. [12], called buffer smoothing algorithms, use the buffers to accept and smooth the variations of bit rate. Studies presented in Jasperneite et.al. [6] and Silvestre.et.al [14] presented another technique called content-based network resource allocation. In these approaches, the latency can be very high since they employ relatively large image buffers in the sender and are based on standard IP networks, using traffic smoothing techniques. In addition, some preliminary processing stages are needed before performing the compression. An increase in latency causes high computational overhead on the sender's side. In video transmission [2], [15] latency comes from video code and network delay or losses. The video coding technique significantly reduces the latency effect of image coding and network delay, which can also be reduced by using RTCP. With these protocols the VBR Streams match with the available bandwidth provided by the communication channels during the transmission. This matching can be done by adopting some parameters [2] like changing frame rate, which results in a frame dropping and low quality frame resolution. To avoid this, we can use some approaches that reserve channel capacity for the maximum bandwidth required by the multimedia content. However, this causes high bandwidth losses with no frame loss. One possible option that can be used to solve this problem is to reserve the channel capacity for the average bandwidth required for the content. This option also causes high network losses/delay, but with efficient utilization of bandwidth.

The main drawback of existing technologies, with regards to their use of industrial communications, is the latency introduced. For example, smoothing video algorithms [19] use memory buffers between the producer and the consumer to smooth the bit-rate variations. Estimates of the required bandwidth and buffering can be handled offline for storing video or, for live (i.e., non-interactive) video streams, can be based on a few images buffered before their transmission. However, the quality of the results depends on the delay allowed by the application. Similar limitations are found in the content-based network resource allocation schemes presented in [7] and [14]. In these approaches, the latency can be high since they employ relatively large image buffers in the sender and are based on standard IP networks, using traffic smoothing techniques. In our proposed model we concentrate on fitting VBR into CBR streams and associated challenges. The QoS model uses the FTT-ESE protocol, which obtains the required bandwidth for real-time multimedia transmission. The goal of this model is to reduce frame loss and utilized bandwidth efficiently with respect to QoS parameters for each stream used in the transmission. Our model takes as input various performance aspects such as allocated bandwidth, compression level, and utilization of network resources for each multimedia source.

Moreover, they require a complementary processing stage before compression in order to adapt the compression to the image content. Thus, the latency is further increased and high computational overhead is incurred by the sender nodes. The latency problem is addressed by the low-delay rate control algorithms [9], [11], [20] that achieve a high performance for videophone and video-conference applications [4], [8], [21], [22].

## System Model

**Figure 1:**
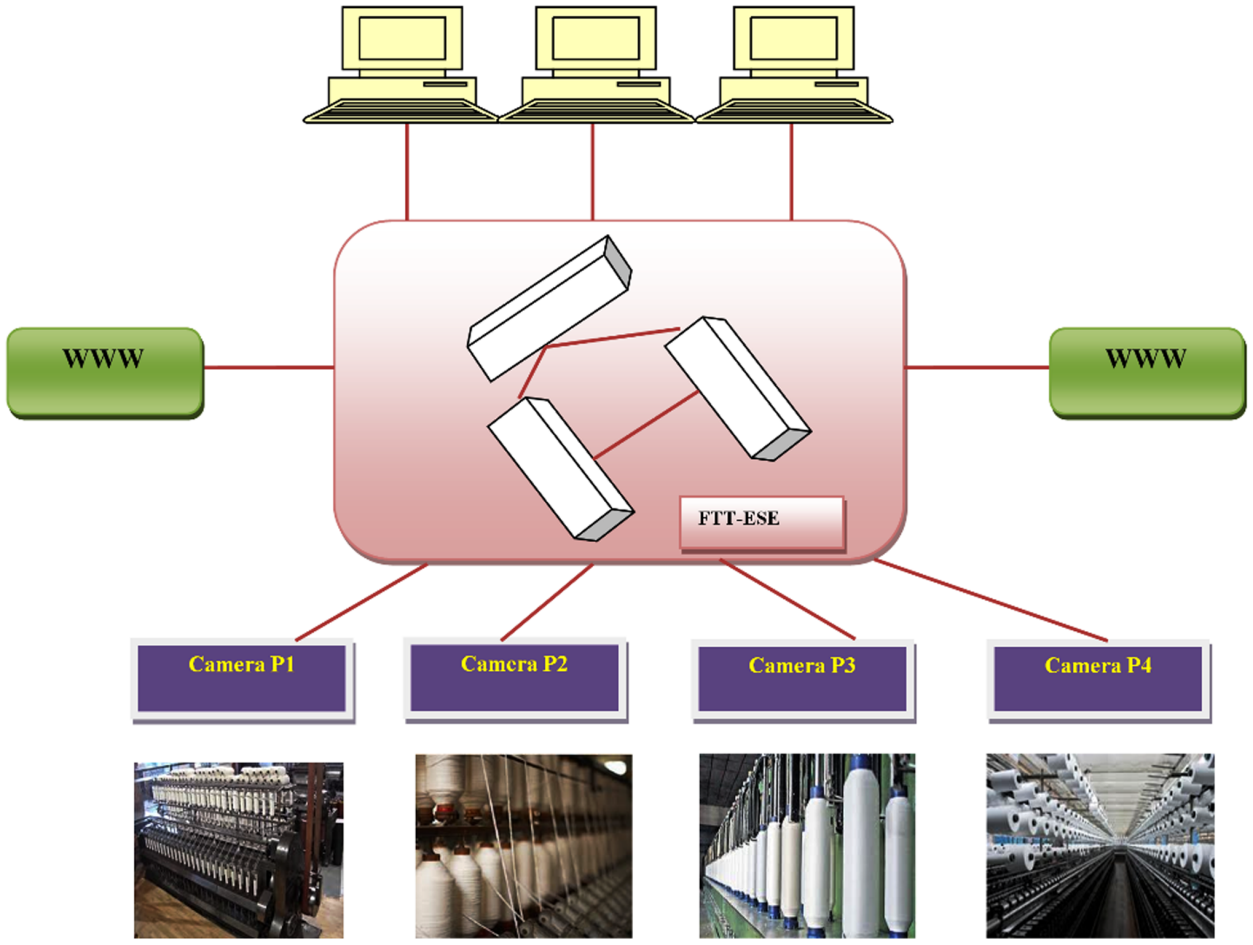
System architecture.

In the proposed work, the sender nodes (multimedia sources) transmit the video streams to multimedia junk, called consumers, via a local area network. Let us define the set of video streams as V =  {V_i_, i = 1,…,n} sent to the receiver nodes. The network should support all other traffic that is aligned with real-time requirements, for example, related configuration or even remote access over the Internet for maintenance purposes (**Fig.1** **).** Due to various communication problems, the proposed model adopts the FTT-ESE Protocol and must resolve all communication requirements. The FTT-ESE protocol is a real-time protocol and possesses the significant features of the fitted model presented in this paper, including QoS management, admission control, and traffic scheduling with synchronous and asynchronous traffic with temporal isolation [2], [5]. FTT-ESE is a master-slave protocol that involves the master node, which holds the message properties, scheduler, admission control, and QoS controller. Slave nodes implement a transmission control layer that controls the network access. The Dynamic QoS model consists of two layers: QoS layer and FTT Layer.

The master node periodically sends the control messages to all the participating slaves, which then carry the message IDs in regular intervals, called as Elementary cycle. The traffic schedule is created by the master nodes for every elementary cycle. The FTT-ESE protocol reserves some portion of the cycle of multimedia traffic. The scenario considered is one in which several nodes send video streams to a junk with a single producer per transmitting node. All nodes remain in the local area network (LAN) and the communication between the sources and the junk is direct.

**Figure 2:**
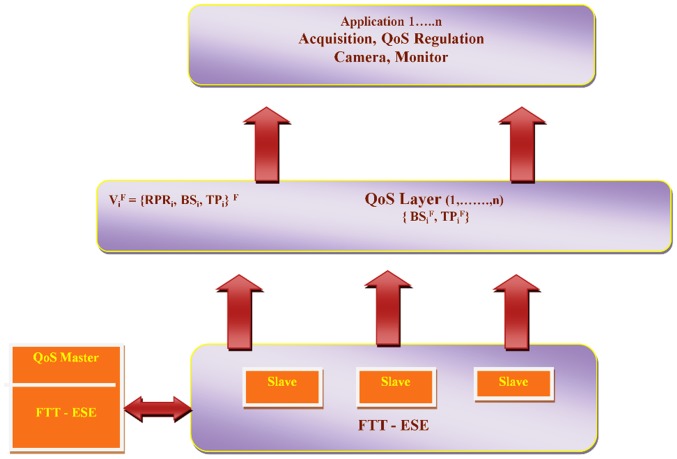
QoS Model.



In **Fig. 2** the scenario is clearly represents the application level in the QoS system characterized the stream by

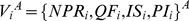
(1)Where NPR_i_^A^ is a stream normalized priority 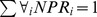
(2)


and *QF_i_^A^  =  {qf_i_^1^,qf_i_^u^*} is the range of compression level qualifying factors, *IS_i_ = { IS_i_^1^, IS_i_^u^*}is the range of frame size after compression and 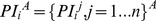
(3)


is a set of interface intervals to the n_i_ allowed rate of the frame.

QoS controller has responsibility to assign bandwidth to each channel receives the channel setup and change requests from the system nodes. Now we consider characterizing the channel for the use of FTT-ESE protocol called as QoS management system.

Let us define the FTT channel parameters by
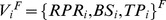
(4)


Where *RPR_i_^ F^* is a relative priority of the channel,

*BS_i_^ F^* is a Buffer size to store the channel, i.e. 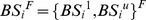
(5)


and *TPi^ F^* is a transmission period of a channel,
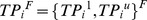
(6)


The output of the QoS Controller is the bandwidth bw_i_ assigned to the channel, buffer size and transmission period. i.e. {*BS_i_, TP_i_*} send again to the QoS layer. Therefore, the bandwidth is
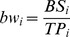
(7)


The responsibility of the QoS layer is to map various QoS parameters of application into various QoS parameters of network. Several QoS layers are adopted in the proposed model. After QoS regulation initializes, the layer begins to map the parameters. At first the maximum buffer size for transmission determined by the layer is 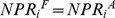 then 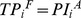 and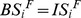. The multimedia stream is fitted to the possible allocated bandwidth by QoS layer. It is achieved by the quantification level factor qf_i_ by discarding unnecessary frames and even deregulated channel bandwidth with the QoS Master node. Let us define the frame bandwidth model by the following equation
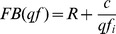
(8)


Where R is a constant of the video stream and C is the compression level value. The frame bandwidth model is used to calculate the quantification level factor for the next frame within the channel target window. The frame will not invoke until the frame bandwidth drops into the channel target window. The frame will drop when the frame bandwidth exceeds the channel width.

**Figure 3:**
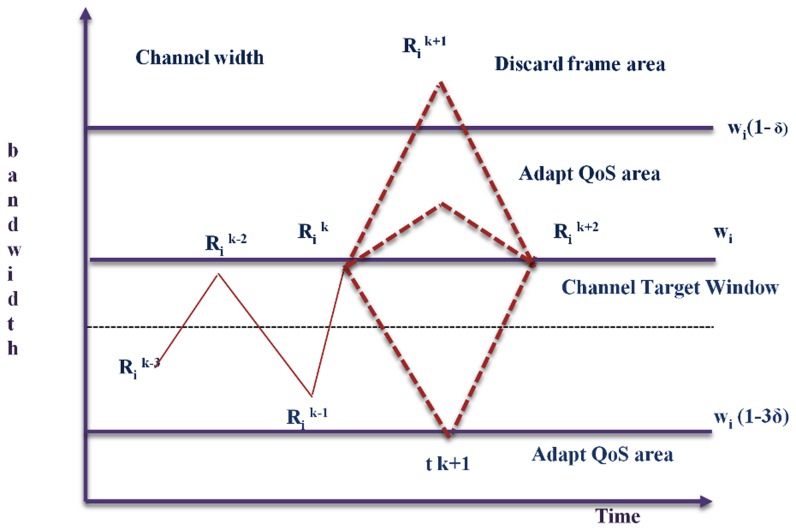
Mapping of VBR into CBR stream.

The frame will invoke at frame bandwidth below the window. The parameter ∂ controls the window, and the results proceed that 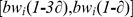 (**Fig. 3**) such that 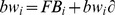, where ∂ is the fractional value of the channels. This value should be between the higher channel bandwidth utilization and lower frequency compression value. The QoS master distributes the network link bandwidth, referred to as V^q^, among the channels according to a predefined QoS policy, the channel QoS parameters, and the current number of active channels. Note that V^q^ is a bandwidth bound that assures the timeliness of the communication channels in each link according to the scheduling policy in use. This bandwidth distribution occurs within the channel renegotiation procedure that is triggered periodically as a response to the online connection/disconnection of streams, to explicit requests from an operator to maximize the QoS of a given stream, or to significant structural changes in any of the active streams. In the first two cases, the bandwidth redistribution is triggered externally, while in the latter case, it is triggered autonomously by a sequence of frames that are dropped or that fall within the channel but above or below the target window. In the dynamic quality of service model, a three-kind of algorithms are performed by the QoS master and slave nodes. The procedure begins at the initiation of the QoS process by an end node. The bandwidth distribution and mapping of bandwidth is carried out by QoS master nodes.

**ALGORITHM: A1- QoS renegotiation**

**Input**: Frame (N_i_)

**Output**: Inverse model of the Frame (P^inv^)

**if** n_i_ > bw_i_

**then**

Qos_variation =  =  P^inv^

upcounter ’ =  =  upcounter+ 1

**else**

upcounter  =  =  0

**if** n_i_ < bw_i_

**then**

Qos_renogotiation  =  =  P^inv^

downcounter  =  =  downcounter + 1

**if** upcounter>QUL or downcounter>QUL

**then**

**return** Qos_variation

The aim of the proposed model is to reduce the number of dropped frames during transmission. Therefore, when the channels are in the channel window or the frame sequence drops within the window, the redistribution of bandwidth is initiated externally. For this condition, we must examine the proposed model with two types of counters, the upcounter and the downcounter, that are used to count the frames above and below the target window. The proposed QoS model recognizes the QoS upper limit value (*QUL*); when the counters exceed this value, the QoS will renegotiate the QoS process. A1 performs the task to evaluate of the QoS variation. A2 performs the task to allocate the bandwidth and A3 performs the task to map the available bandwidth to FTT channel parameters, i.e.(*BS_i_,TP_i_*).QoS layer adjusts the computational quantifying factor for each frame in a stream. QoS master adjusts the computational factor, when the factor drops in the out of the range of the QoS layers. When the computational factor is not able to set up the stream bandwidth to the channel window then the frame dropped. The QoS variation has admitted when more than the computational factor generated. This occurs due to the changes in the frame and some QoS parameters involved in the application. For QoS renegotiation, the proper bandwidth for each stream bw_i_ is calculated. Now bw_i_ is compared the link bandwidth with the QUL values. If it is not enough, then the bandwidth allocation algorithm used to find the efficient bandwidth (bw_i_) for each stream. Finally the system calculates the bandwidth of each stream translated into FTT computational parameters (*BS_i_, TP_i_*) (algorithm A3).

Algorithm A2 is based on the constant priority that is carried out on this proposed model. First, it estimates the minimum bandwidth (*BW_i_^min^*) and allocates the remaining bandwidth to all channels with priority order (*NPR_i_*); it is possible that in all-time, most of the channels will not receive enough system bandwidth. Thus, some channels will receive the requested bandwidth and the remaining channels will receive the average value of the bandwidth. Algorithm A3 performs the bandwidth mapping with the FTT channel parameters for transmitting stream. Note that there are many different possibilities to carry out both the bandwidth distribution and the mapping of stream bandwidth onto network parameters. The algorithms presented here and explained next are just one possibility that, nevertheless, is effective. Performing an extensive analysis and comparison of different algorithms for these purposes are out of the scope of this paper. The bandwidth allocation algorithm sets the bandwidth (*BW_i_^qos^*) to all channels. Now *BW_i_^qo^* ^s^ is converted into the (*BS_i_, TP_i_*) duplet used by the QoS and FTT layers; it is possible that different (*BS_i_, TP_i_*) pairs may be produced in the same bandwidth.

**ALGORITHM: A2 - Bandwidth Distribution**

**Remarks: Distributed the system bandwidth capacity**

**for each** Vi ∈ V sorted by NPR_i_

**do**

if(bw_i_ – bw_i_^min^) < network link bandwidth

**then**

bw_i_  = bw_i_ – bw_i_^min^

**else**

bw_i_’ =  network link bandwidth

**return** BW_i_^QoS^  =  { BW_1_^QoS^,……., BW_n_^QoS^}

Equation (9) satisfies the mapping algorithm; this should not exceed the bandwidth assigned by algorithm A2. The FTT level is BS_i_ bound first and then identifies TP_i_. Many mapping algorithms consider performing mapping, but the alternative that is selected depends on the application.
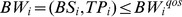
(9)


The transmission period (TP) is computed by an algorithm mapping to the assigned bandwidth BW_i_^qos^ with BS_i_^1^ = BS_i_^u^, where BS_i_^u^ is an upper bound of bandwidth distribution. If the period has discrete values, we choose the closest value, but with the least value in the TP_i_^FTT^, we can have an estimate that leads to a greater bw_i_ than was assigned 
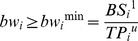
(10)


where 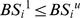 implies that 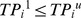. With equation (10), we clearly understand that we can choose the next transmission period in the list when the condition is satisfied. Finally, note that Algorithm 1 (excluding the QoS renegotiation) that executes in the end nodes, as well as both Algorithms 2 and 3 that execute in the master node, incurs on a negligible computation overhead that, in a common PC hardware, may represent, at most, a few microseconds.

The result of the bandwidth distribution is the set of channel bandwidth assignments (*BW_i_^qos^*) for all channels in the system. However, the bandwidth itself is not an operational parameter. Consequently, it must be converted into a (*BS_i_, TP_i_*) duplet to be used by the QoS and FTT sub layers. This conversion is not univocal since different (*BS_i_, TP_i_*) pairs may produce the same bandwidth. Furthermore, at the FTT level, BS is bounded, and TP may have restrictions, e.g., due to the need to match camera frame-rate restrictions, thus, a direct correspondence between *BW_i_^qos^* and a (*BS,TP*) pair may or may not exist. In this case, the mapping algorithm has to compute a bandwidth value that approaches, without exceeding, the bandwidth granted by the bandwidth distribution algorithm

(i.e., 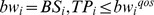). Several mapping approaches are possible, and choosing the best one is application dependent. Algorithm 3 describes a mapping approach that attempts to maximize the transmitting budget *BS_i_*, bringing it as close as possible to the application desired upper value *BS^u^_i_*, while keeping the allocated channel bandwidth *BW_i_.* To do so, first, the algorithm computes the period TP that corresponds to the allocated bandwidth BW_i_^qos^ *with BS_i_ =  BS^u^_i_*_._ Since the periods are discrete, we use the closest, but lower value in the monotonically increasing set *TP_i_^FTT^*. This approximation eventually leads to a bandwidth bw_i_ that can be greater than the allocated one.

**ALGORITHM: A3 –Mapping of (BS_i_, TP_i_) with (BW_i_, V_i_)**

**for each** V_i_ ∈ V

TP_i_ = max{ TPi^1^…..TPi^n^}

BS_i_  =  BW_i_^QoS^ × TP_i_

**if** BS_i_ < BS_i_^u^

**then**

TP_i_  =  desc(TP_i_)

BS_i_  =  BS_i_^u^

**return** ((BS_i_, TP_i_) ∀_i__ ,_ {bw_1,………_ bw_n_})

In such cases, BS_i_ is recomputed to match the allocated bandwidth. However, in the sequel, it may happen that the computed BS_i_ violates the defined lower bound (*BS_i_ < BS^l^_i_*). In that case, the next value in the period list succ(*TP_i_*) is selected, and *BS_i_* is made equal to *BS^u^_i_,* which means that an exact bandwidth match cannot be found resulting in a reduced bandwidth w_i_. Finally, note that, as long as 
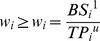
 implies *BS_i_ < BS^l^_i_* that *TP_i_ < TP_i_^u^*, and thus, there will always be succ(*TP_i_*) in the set in that case.

## Results and Discussions

**Figure 4:**
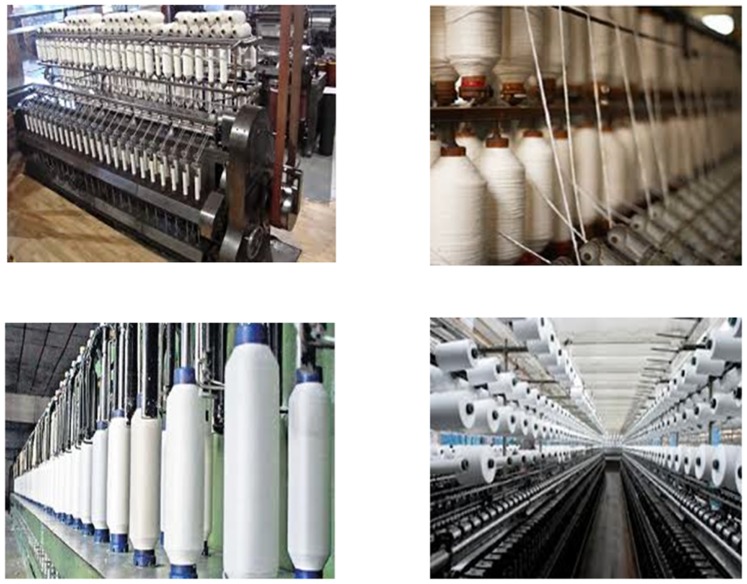
Example of images in the streams used.

**Figure 5:**
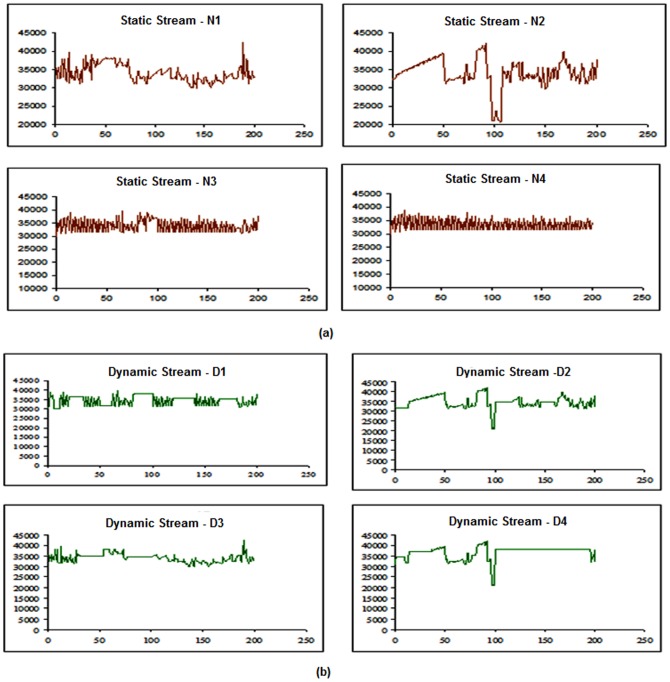
Frame size evaluation. **a)** Static frame size evaluation. **b)** Dynamic frame size evaluation.

To evaluate the performance of the proposed model presented in this paper, several experiments were carried out using the streams described in **Table 2**. **Fig.4** presents some frames of those streams. To validate the solution we have designed and implemented a simulator based on the Matlab/Simulink which is a tool to evaluate different network architecture using Simulink blocks. All of the obtained streams have r_i_  =  560×412 pixels, *T_i_*  =  40*ms*, and are 4000 frames long (160 seconds).This set of streams is based on those presented in [16], consisting of pre-recorded sequences obtained in industrial environment, namely, the textile industry environment (TF). Four different streams, namely (N1, N2, N3, N4) and (D1, D2, D3, D4) have different static and dynamic changes in the real-time as shown in the **Fig. 5a** and **Fig. 5b**. It shows the evolution of the frame size in each stream compressed with a constant *q*, showing its dynamics, complexity, and requirements. (N1, D1) and (N4, D4) stream represents the rapid change bandwidth and (N2, D2) and (N3, D3) streams labelled as dynamically changed parameters. Two groups of testing carried out in the proposed model, the first one is based on the dynamic streams and the second one is based on the static stream properties (not much more frequent changes in the streams).The quality of the video has measured in the various categories like quality of the frame and the stream properties. These metrics considered in to the account of degrade the image, but not for the bandwidth utilization. The new parameter introduced for each stream for efficient use of the bandwidth. Experiments dt1-dt3 use *C*  =  48 kB and *T*  =  60 ms, resulting in a total bandwidth of 32.4 Mb/s, with quantification factors set to 40, 45, and 50, respectively. In experiment dt4, *C* was set at 28 kB, and *T* was set to 30 ms, yielding a similar bandwidth, while the quantification factor *q* was set to 25.For calculating QoS priority to identify the lower waste of bandwidth from the number of frames dropped from each stream N_i_ is given below

**Table 2:** Stream properties.

dt1-dt4	N1/TF1	N2/TF2	N3/TF3	N4/TF4
qf_1_	30	45	30	25
qf_u_	60	60	65	58
bw_1_	40k	40k	20k	20k
bw_u_	60k	60k	50k	50k
fp_1_ (ms)	45	45	45	45
fp_u_(ms)	140	100	100	140
PR_r_	0.172	0.172	0.172	0.172

The global QoS is also computed as the average of the QoS_i_ parameters. In the following experiments, we will use the classic noise ratio tip Signal (NRTS) and index of quality (IQ) to characterize the quality of each individual stream and the QoS metric for assessing the aggregated QoS of each experiment. Noise ratio tip Signal (NRTS) is one of the quality metric most frequently used to evaluate the performance of codecs and video transmission systems.



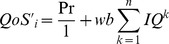
(11)


In most of the streams, the increase in quality compensates the higher number of dropped frames.N3 is a stream having lower dynamics, reducing δ actually improves the NRTS metric, although slightly, since the sequence not affected by dropped frames. To assess the impact of the QoS management techniques, a several global QoS metrics that compare the streams with the raw original ones, frame by frame. The received image quality reviewed with the NRTS; [measured in decibels], as well as with the index of Quality (IQ) which believed to provide a better correlation with human perception than the NRTS. Video quality usually calculated using the frame quality average. Being *f* the original image, and *g* the distorted one, the image quality index (IQ) [12] can be calculated as:
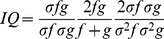
(12)


where *f* and *g* are the intensity mean, σ*f* and σ*g* its variance and σ*fg* the covariance. The range of values for the *g* index Q is [−1, 1], being Q = 1 when the images are identical.Usually, these metrics consider for average image degradation only, ignoring the efficiency of the channel bandwidth utilization. In this, a new metric that weighs each stream with its priority and accounts for the efficient use of the channel bandwidth by favoring the streams that present lower bandwidth waste. In the following tests, IQ and NRTS characterize the quality of each individual stream and QoS metric to assess the aggregated QoS for each test. For each test, the **tables 3** **and** **4** shows the number of dropped frames (*DrF*), the wasted bandwidth *WB* (measured in megabits per second), and the quality according to the NRTS and IQ criteria.

**Table 3:** Simulated dynamic streaming experimental results.

dt 1	Dropped Frames (DrF)	Wasted Bandwidth (wb)	Noise Ratio Tip Signal (NRTS)	Index of Quality (IQ)
D1	9	0.78	32.7	0.82
D2	0	1.00	34.7	0.97
D3	4	0.67	32.1	0.79
D4	7	0.81	33.4	0.80
median	5	0.815	33.225	0.845

dt 2	Dropped Frames (DrF)	Wasted Bandwidth (wb)	Noise Ratio Tip Signal (NRTS)	Index of Quality (IQ)
D1	15	0.30	32.3	0.82
D2	08	0.38	33.9	0.84
D3	17	0.42	32.1	0.81
D4	12	0.35	33.2	0.80
median	13	0.3625	32.875	0.8175

dt 3	Dropped Frames (DrF)	Wasted Bandwidth (wb)	Noise Ratio Tip Signal (NRTS)	Index of Quality (IQ)
D1	6	0.88	32.5	0.88
D2	0	0.87	35.2	0.90
D3	5	0.78	33.2	0.87
D4	9	0.76	34.2	0.86
median	5	0.8225	33.775	0.8775

dt 4	Dropped Frames (DrF)	Wasted Bandwidth (wb)	Noise Ratio Tip Signal (NRTS)	Index of Quality (IQ)
D1	24	0.41	33.4	0.85
D2	4	0.40	31.2	0.89
D3	36	0.34	33.7	0.84
D4	26	0.39	32.2	0.86
median	22.5	0.385	32.625	0.86

**Table 4:** Simulated static streaming experimental results.

Static test 1	Dropped Frames (DrF)	Wasted Bandwidth (wb)	Noise Ratio Tip Signal (NRTS)	Index of Quality (IQ)
N1	6	1.12	29.4	0.86
N2	2	1.45	32.9	0.84
N3	9	0.59	29.78	0.85
N4	7	1.23	31.2	0.83
median	6	1.0975	30.82	0.845

Static test 2	Dropped Frames (DrF)	Wasted Bandwidth (wb)	Noise Ratio Tip Signal (NRTS)	Index of Quality (IQ)
N1	12	1.44	30.55	0.82
N2	2	1.18	30.82	0.85
N3	24	0.64	30.02	0.83
N4	18	0.78	30.73	0.81
median	14	1.01	30.53	0.8275

Static test 3	Dropped Frames (DrF)	Wasted Bandwidth (wb)	Noise Ratio Tip Signal (NRTS)	Index of Quality (IQ)
N1	4	0.92	34.4	0.89
N2	43	0.54	32.3	0.85
N3	46	0.62	32.07	0.86
N4	32	0.78	31.5	0.84
median	31.25	0.715	32.5675	0.86

Static test 4	Dropped Frames (DrF)	Wasted Bandwidth (wb)	Noise Ratio Tip Signal (NRTS)	Index of Quality (IQ)
N1	11	1.01	30.11	0.81
N2	1	1.61	31.23	0.85
N3	9	1.13	30.27	0.83
N4	15	1.21	30.54	0.84
median	9	1.24	30.5375	0.8325

Reducing δ causes a consistent drop on the wasted bandwidth, as expected. However, this drop achieved at the expense of an increased number of dropped frames. This effect is visible in streams that show higher dynamics. For streams that have more stable requirements, the impact is minor or even null. The impact on the number of dropped frames is, however, not always reflected in the image quality metrics. Adjusting δ causes the increment of dropped frames and increases the bandwidth of the window which makes the higher efficiency of using the width of the channel, gives the entry to quantify the QoS adaptation layer compression measures. In all streams testing, when the higher number of frames drop then the quality increases. In **Fig.5a** and **Fig.5b**, the Y axis shows the frame size value and X axis shows the constant q value.

**Tables 3** **and** **4** report the experimental results obtained from the test. For streams with lower dynamics, reducing δ improves the NRTS metric, although slightly, the sequence affected by dropped frames. Comparing **Tables 3** **and** **4** clearly show that the dynamic approach leads to significant improvements in all key aspects. The streams with high dynamics, the dropped frames are reduced. The quality parameters like IQ and NRTS are averagely good. The utilization of bandwidth also improved quietly. It should be remarked that these results are achieved with better bandwidth utilization.

**Figure 6:**
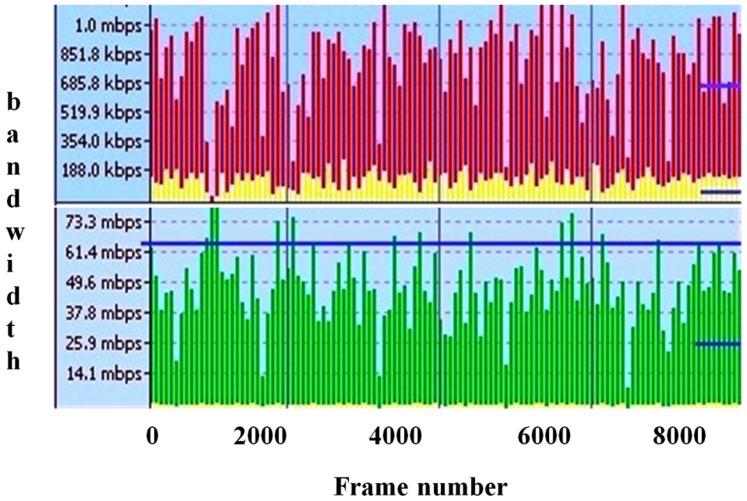
Stream N1 bandwidth evolution.

**Figure 7:**
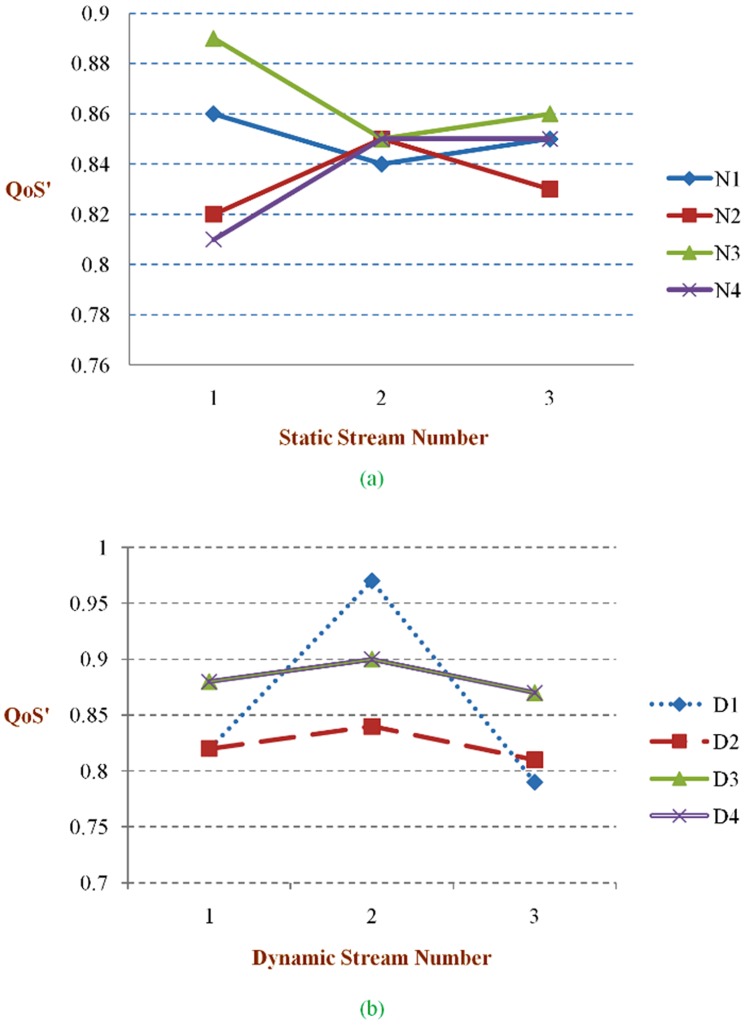
QoS contribution. **a)** Static stream to QoS'. **b)** Dynamic stream to QoS'.

**Figure 8:**
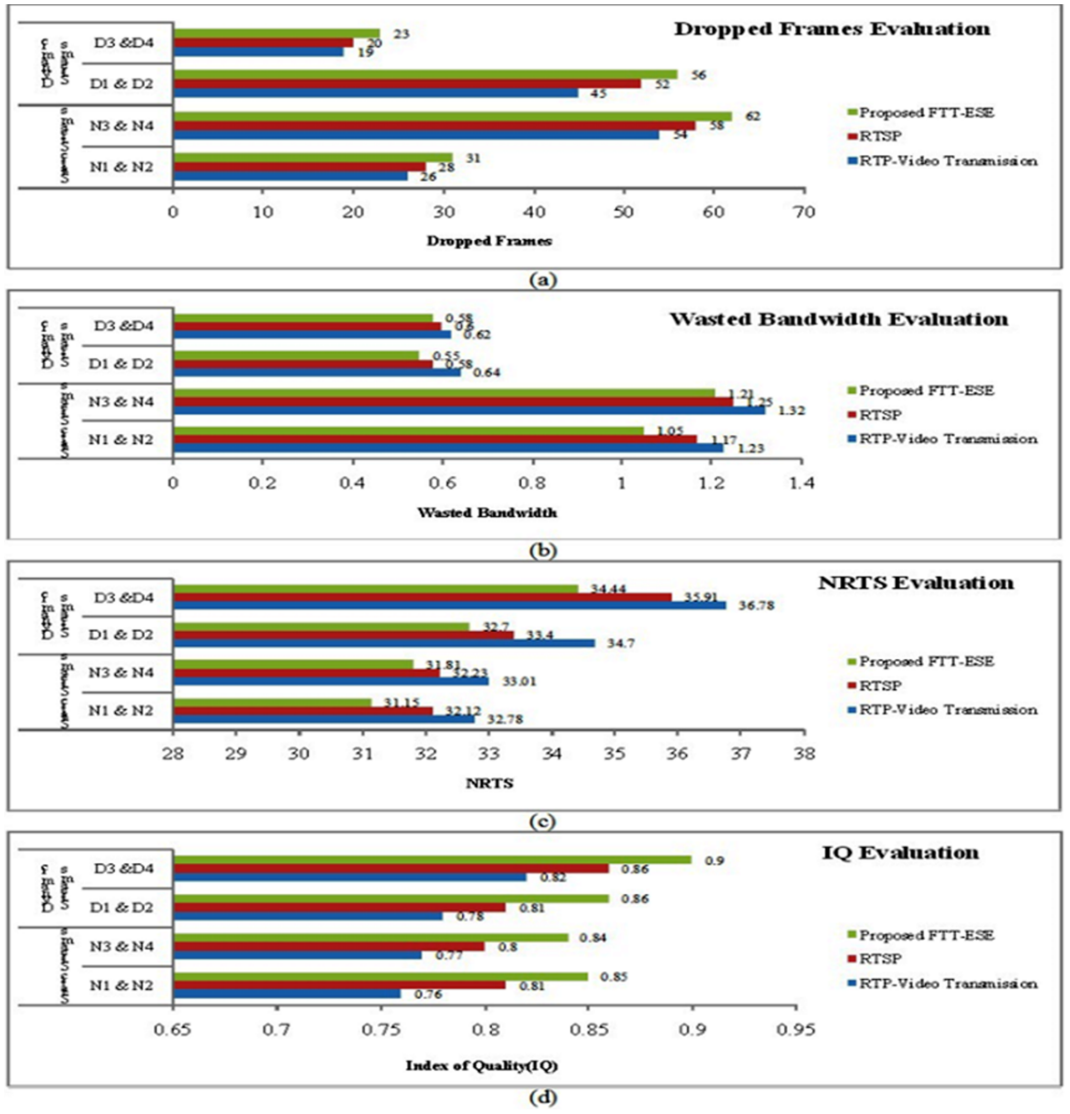
Comparison of static and dynamic streams of various frameworks with FTT-ESE. **a)** Dropped frames (DrF). **b)** Wasted bandwidth (WB). **c)** Noise ratio tip signal (NRTS). **d)** Index of quality (IQ).

**Table 5** presents the QoS' values for each test. The first conclusion that can be drawn is that, for properly selected *δ* parameters, the QoS reached with the dynamic approach may be significantly higher than that reached with the static approach. Considering the meaning of this metric, one may conclude that higher quality levels may be attained both by allocating more bandwidth to the streams that can make better use of it, as well as by reducing the wasted bandwidth. The impact of the wasted bandwidth in this metric may also be observed in the significant difference, around 45%, between experiments dynamic test1 (dt1) and dynamic test2 (dt2), and between dynamic test3 (dt3) and dynamic test4 (dt4). **Table 6** shows the combined comparison of the various key elements gained by the proposed model. **Fig. 6** shows the bandwidth used by stream (N1, D1) in experiments dt1, dt2, and dt4. It can be observed that, in experiment dt4, the scheduler assigns more bandwidth to stream (N1, D1) than in experiments dt1 and dt2. This observation is particularly clear when comparing experiments dt2 and dt4, which have an equivalent parameterization except for the priority. Observing **Table 3**, it is possible to conclude that the higher priority streams have a gain between 0.7 and 1.5 dB, at the expense of a decrease between 0.8 and 1 dB in the lower priority ones. Thus, the priority mechanism proves its effectiveness in differentiating the streams, providing more resources to the ones that have higher impact in the global system performance. **Fig. 7a** **and** **Fig. 7b** shows the various IQ levels for static and dynamic streams. The IQ is high in the static and dynamic streams, as shown **Table 6**. Experiment dt4 aims to illustrate the system behavior when the assigned priorities are non uniform. The Pr values used in dt4 imply a bandwidth distribution where streams (N1, D1), (N3, D3) and (N4, D4) obtain more resources in detriment of stream (N2, D2) as can be seen in **Fig. 7b**. This matches the requirements of many applications in which some streams have a higher impact on the global system performance and thus should be favoured. **Fig. 8a** **–** **Fig 8d** presents the clear outcomes of the proposed model with static and dynamic streams and also it shows the higher bounds of the proposed model with the QoS parameters.

**Table 5:** QoS results.

QoS'	N1	N2	N3	N4
	0.85	0.83	0.86	0.83

QoS'	D1	D2	D3	D4
	0.85	0.82	0.86	0.88

**Table 6:** Comparison of enhanced QoS model parameters.

QoS Parameters	Static Streams		Dynamic Streams	
DrF	N1 & N2	N3 & N4	D1 & D2	D3 &D4
RTP-Video Transmission	26	54	45	19
RTSP	28	58	52	20
Proposed FTT-ESE	31	62	56	23

WB	Static Streams		Dynamic Streams	
	N1 & N2	N3 & N4	D1 & D2	D3 &D4
RTP-Video Transmission	1.23	1.32	0.64	0.62
RTSP	1.17	1.25	0.58	0.60
Proposed FTT-ESE	1.05	1.21	0.55	0.58

NRTS	Static Streams		Dynamic Streams	
	N1 & N2	N3 & N4	D1 & D2	D3 &D4
RTP-Video Transmission	32.78	33.01	34.7	36.78
RTSP	32.12	32.23	33.4	35.91
Proposed FTT-ESE	31.15	31.81	32.7	34.44

IQ	Static Streams		Dynamic Streams	
	N1 & N2	N3 & N4	D1 & D2	D3 &D4
RTP-Video Transmission	0.76	0.77	0.78	0.82
RTSP	0.81	0.80	0.81	0.86
Proposed FTT-ESE	0.85	0.84	0.86	0.90

One aspect that should be highlighted is the low sensitivity of the system to the particular values of δ and QUL. In fact, the NRTS and IQ metrics do not change significantly with any of these parameters, thus facilitating system setup. The number of dropped frames is strongly reduced in the streams with higher dynamics (e.g., (N1, D1) and (N4, D4)). The quality metrics (NRTS and IQ) are also consistently similar or better. It should be remarked that these results are achieved with better bandwidth utilization In fact; it is possible in some cases, to find the best static q for each stream. However, this procedure has to be done offline, thus not being suitable for multimedia embedded system applications. Finally, note that the use of pre recorded video sequences instead of cameras was transparent operation of the system and that, as expected, no performance bottleneck was found despite the frequent QoS adaptations (adaptations of q) and occasional channel bandwidth renegotiations to the operation of the system and that, as expected, no performance bottleneck was found despite the frequent QoS adaptations (adaptations of q) and occasional channel bandwidth renegotiations.

## Conclusions

The proposed QoS model proves that it is possible to change the channel bandwidth dynamically according to the streams and the available bandwidth. This proposed model is extensively assessed, with its performance compared against a similar situation with static CBR channels, using a set of stored video sequences from industrial environments. The primary network should support the multimedia streams in real-time applications. The system has to reduce the wasted bandwidth for increasing performance of the multimedia streams. Instead of using VBR channels, the system adapts with the CBR channels to increase the performance of the system. A new QoS metric that considers the image quality, stream priorities, and the capacity of the system to reduce wasted bandwidth is used to assess the performance. The results obtained show a consistent superiority of the dynamic adaptation mechanism, when there are streams of different priorities. In the future the real-time streams from different industries will adopt with this model and to increase the stream properties with different set of Quality parameters have to analyze and will increase the Quality.
